# Application of Polymer Drugs with Cerium Dioxide Nanomolecules and Mesenchymal Stem Cells for the Treatment of Skin Wounds in Aged Rats

**DOI:** 10.3390/polym13091467

**Published:** 2021-05-01

**Authors:** Ekaterina Vladimirovna Silina, Victor Aleksandrovich Stupin, Yulia Gennadievna Suzdaltseva, Salekh Rovshanovich Aliev, Igor Sergeevich Abramov, Nikolay Valerievich Khokhlov

**Affiliations:** 1 Department of Human Pathology, I.M. Sechenov First Moscow State Medical University (Sechenov University), 119991 Moscow, Russia; 2 Department of Hospital Surgery No. 1, N.I. Pirogov Russian National Research Medical University (RNRMU), 117997 Moscow, Russia; stvictor@bk.ru (V.A.S.); qwe1.80@mail.ru (S.R.A.); abramov-1961@mail.ru (I.S.A.); Nikolay.khokhlov@gmail.com (N.V.K.); 3 Department of Epigenetics, Vavilov Institute of General Genetics of the Russian Academy of Sciences, Gubkin str. 3, 119991 Moscow, Russia; yu_suzdaltseva@mail.ru

**Keywords:** smart polymeric nano-drug, polymer, cerium dioxide, stem cells, wound healing, healing of skin wounds

## Abstract

The urgency of the problem of wound healing is not in doubt, given the global trend of an increase in the number of operations and injuries with skin damage, as well as the lack of universal means of treating wounds. Study Objective: To compare the effectiveness of the developed drugs, smart polymeric nano-drug with cerium oxide nanoparticles (SPN), and smart polymeric nano-drug in combination with mesenchymal stem cells (SPN + SC) on the healing process of skin wounds. Material and methods. An experimental study was carried out using Wistar rats of post-reproductive age, which had dermis and epidermis removed on their backs. There were four groups of wounds in total: control, treatment with mesenchymal stem cells (SC), SPN, and SPN + SC. Results. A positive therapeutic effect of polymeric drugs on the dynamics of wound area reduction was established, which was most typical for wounds of the SPN group and, particularly, the SPN + SC group. On the third day, an anti-inflammatory effect was revealed in the SC and the SPN + SC groups in particular, which was expressed in a reduced leukocyte infiltration and an increase in the level of microcirculation during this period. The fastest transition from the phase of exudation to proliferation was recorded in the SPN and SPN + SC groups. Histologically, these groups showed faster regeneration, including the epithelialization of wounds. Conclusion. The results obtained in the course of the study open up possibilities for the development of fundamentally new, highly effective wound healing agents.

## 1. Introduction

Medicine, as a science, began to form before our era, and even then, taking into account the incessant wars, its main task was to treat wounds. It all started with plant products (infusions, decoctions, etc.). However, as the world population and the number of wounded increased, the area for sowing medicinal herbs and the development of chemical production decreased, which led to a switch to cheaper synthesized medicines. To date, thousands of drugs have been synthesized; however, the percentage of unsatisfactory results in the treatment of acute and especially chronic wounds remains high [1,2,3]. This can be explained by the fact that practically all modern medicines act only on one of possibly many pathogenetic mechanisms of the appearance and development of the disease. Unfortunately, there is still no universal medicines that immediately affect all links in the pathogenesis of various wounds or change their structure depending on the biochemical characteristics of the wound [4,5]

The last half-century has seen the rapid development of biology and a deeper understanding of the processes that control cell life and death. One of the areas gaining more supporters is the study of the possibility of using stem cells in clinical medicine to stimulate the healing of skin wounds and burns [6,7,8,9]. Stem cells (SC), or progenitor cells, are undifferentiated mesenchymal cells, capable of differentiating into any soma cells upon death or damage to any tissue structures. Hundreds of studies have shown the positive effect of both self (auto-) and allogeneic SC, confirming the preliminary hypothesis of the enormous importance of these cells for any organism in the restoration of tissue damaged as a result of disease or external influences. However, the cultivation of such cell cultures and their accumulation requires large capital investments and significant costs in the process of their cultivation and storage. In addition, at the beginning of differentiation into any type of somatic cell, specific proteins appear on its surface in an allogeneic cell—cellular markers that trigger the mechanism of the recipient’s immune response—and the cell is destroyed. The creation of an autocellular culture of SC for a particular patient is a long process. Although it brings the arrival of personalized medicine closer, it is extremely expensive. In addition, no breakthrough in wound or burn healing has been achieved in any of the studies conducted. All of this drives the search for new chemicals capable of accelerating the processes of tissue regeneration.

The search began on organic or inorganic substances with an unknown effect on the cells of micro- or macro-organisms. In recent years, research data have begun to accumulate on the positive role of various polymers on wound healing [10,11,12,13,14,15]. In addition, cerium dioxide was found to be capable of exhibiting antioxidant properties in a cell culture or wound due to the ability to change its valence and stabilize the pH of the intercellular and wound environment [16,17,18,19]. This gives us reason to call such substances smart nano-drugs. Scientists from different countries have demonstrated the effectiveness of using cerium oxide nanoparticles for healing skin wounds [20,21,22,23,24,25]. Since the theory of oxidative stress as the main mechanism of cell death and the occurrence or maintenance of non-healing wounds is gaining increasing confirmation, the researchers decided to study the effect of these agents (factors) on the healing of experimental acute wounds in animals.

The aim of the study is to compare the effectiveness of the developed drugs, smart polymeric nano-drugs (SPN) alone and smart polymeric nano-drugs in combination with stem cells (SPN + SC) on the healing process of skin wounds.

## 2. Materials and Methods

This study involved 56 male Wistar rats 36–40 week-old (median: 38 weeks) weighing an average of 419.7 ± 42.3 g. After 2 weeks of quarantine (the animals were kept in standard vivarium conditions with free access to food and water), the rats were randomized into four groups, differing in the treatment of wounds. The next day, square wounds were simulated on the animals’ backs on either side of the spine using a patented device that allows for the creation of skin wounds of a standardized size (Patent RF 79701/10.01.2009). The operation was performed under general anesthesia (intraperitoneal chloral hydrate 300 mg/kg) in non-sterile conditions. A total of 112 wounds were modeled. The area of the wounds, according to estimates, 10–20 min after their modeling, on average in all rats was 138.0 ± 12.2 mm^2^, and the depth of the wounds was down to the muscle fascia, removing all layers of the epidermis and dermis. We used animals not young, but of mature age, because the problem of wound healing is most acute in people in the second half of life.

Wound simulation day was study day 0. Immediately after modeling the wounds, the animals were housed in individual cages until the endpoint of the study. The study of the dynamics of wound healing was performed on days 1, 3, 5, 7, and 14. The timing at which the animals were removed from the experiment was 3, 7, and 14 days. The animals were removed from the experiment under general anesthesia (chloralhydrate 300 mg/kg, intraperitoneal) by rapid exsanguination of the rat by opening the right ventricle of the heart.

The manuscript presents the results of the treatment of four groups of wounds. Initially, there were 28 wounds in each group.

1. Control group—these are intact wounds without treatment.
2. Group of mesenchymal stem cells (SC)—a culture of mesenchymal stem cells at a concentration of 100,000 SC per wound) was injected into the edges of the wounds of this group on the modeling day (0.2 mL: 0.1 mL of SC was injected into the lateral edge, and 0.1 mL of SC into the caudal edge). SC were isolated from the umbilical cord of a newborn after normal delivery at 38–40 weeks of gestation with the informed consent of a healthy mother and produced after the fourth passage by technology [25,26]. After the isolation process, the stem cells had a standard morphological structure of cells grown in a MEM culture medium (Lonsa), StemPro ™ MSC (Gibco, Grand Island, NY, USA), at a concentration of 0.5 × 10^6^ per 1 mL.
3. Smart polymeric nano-drug (SPN) group—citrate-stabilized cerium dioxide nanoparticles were integrated into a hydrogel polymer matrix. The cerium dioxide used in this work was synthesized at the Kurnakov Institute of General and Inorganic Chemistry of the Russian Academy of Sciences (Moscow) [27,28]. The hydrogel matrix contained natural and synthetic polymers of structure-forming polysaccharides (pectin, alginate, chitosan, agar–agar, and water-soluble cellulose derivatives, including carboxymethyl cellulose). To prepare the hydrogel base of the nano-drug in 975 mL of H_2_O, we added a dry sample of carboxymethylcellulose 20 g, pectin (Sigma Aldrich) 2 g, chitosan (Sigma Aldrich) 0.2 g, agar–agar (Roeper) 2 g, and sodium alginate (Sigma Aldrich) 1 g. Next, a colloidal sol with a cerium oxide concentration of 0.004 g/l was added dropwise to 25 mL of the prepared gel matrix with constant stirring. As a result, the concentration of substances in the gel base of the matrix was (in mass percent): carboxymethyl cellulose—1.0; pectin—0.1; chitosan—0.01; agar—0.1; alginate—0.05; cerium oxide in the form of a colloidal sol of nanoparticles—0.0002. A preliminary analysis of the developed agent, as well as the results of experiments, demonstrating its safety and biocompatibility, were presented earlier [24,25,29,30]. SPN was applied topically once a day on day 0, 1, 3, 5, and 7 by applying it over the wound with a needle-free syringe and filling its volume using 1 mL per cm^2^.
4. Smart polymeric nano-drug + stem cells group (SPN + SC)—this differed from the SPN group only in that, in addition to cerium dioxide, mesenchymal stem cells of the human umbilical cord at a concentration 50,000 cells/cm^2^, produced using the same technology as for the SC group, were also added to the polymer matrix. The application of SPN + SC was carried out non-invasively by surface application of hydrogel days until the entire volume of the wound was filled (as in the SPN group for the same technology and at the same time).

Hydrogel polymer preparations (SPN and SPN + SC groups) were carefully applied to the wound until it was completely filled. Within 10–20 min, a biomembrane formed on the surface of the polymeric nano-drug, which reliably closed the wound and created a good environment for the preservation of stem cells in the SPN + SC group. Thus, there was no need for patches. We did not use surface dressings in any groups.

The wound healing process was assessed by a set of indicators.

The area of the wounds was examined at 0, 1, 3, 7, and 14 days. First, the wounds were photographed (Canon EOS550D digital camera (Canon, Tokyo, Japan) with a Canon EF-S18-55 lens (Canon, Tokyo, Japan) mounted on a tripod (focal length of 50 mm, Jpeg digital image format) at the same angle and at a distance to the wound of 30 cm. The same ruler was used throughout the experiment on all rats. The wound area was calculated from the photographs using the JMicroVision 1.2.7 software (Geneva, Switzerland), expressing the final result in mm^2^.

Microcirculation was studied in anesthetized rats on days 0, 3, 7, and 14 (twice in each animal: on the day of simulation and on the day of hatching). For this, the MP150 hardware–software complex for electrophysiological studies (BIOPAC Systems, Inc., Goleta, CA, USA) with a module for laser-Doppler flowmetry LDF100C and the AcqKnowledge version 4.4.1 program was used. The complex detects the presence of a frequency shift of the reflected signal and, in accordance with the software signal processing algorithm, converts them into perfusion units (blood perfusion units, BPU). The needle sensor was installed perpendicular to the skin, 2 mm outward along all four edges of the wound. The end result was the arithmetic mean of the BPU obtained from all edges of the wound.

**Figure 1:**
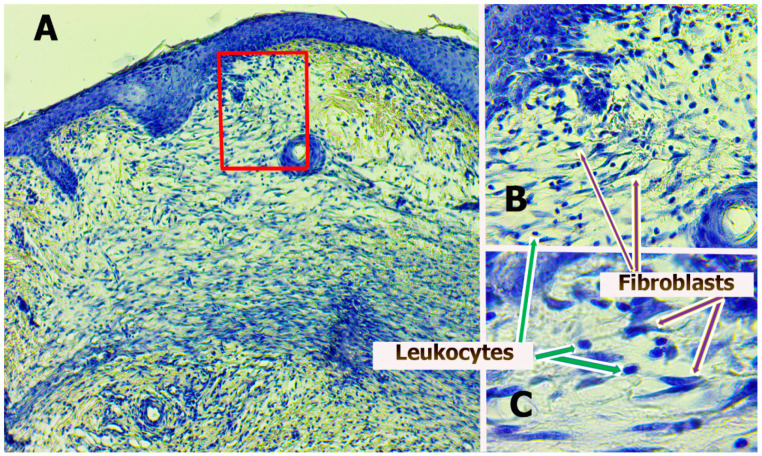
Edge of wounds, group SPN + SC on Day 7. Leukocytes (round cells) and fibroblasts (elongated cells with elongated nuclei, they produce the intercellular substance) are visualized. Hematoxylin. (**A**). Magnification ×100. (**B**). Magnification ×400. (**C**). Magnification ×1000.

The morphometry and microscopy of wounds were performed after hatching the animals on the 3rd, 7th, and 14th days, as was the preparation of histological sections of wounds, stained according to different methods. For each wound, at least three consecutive sections were made. Hematoxylin–eosin staining was performed for descriptive light microscopy. Staining with hematoxylin alone was carried out for processing sections in the Image-J program (National Institutes of Health, Bethesda, MD, USA) with a quantitative analysis of the number of non-resident cells (cells migrating to the lesion focus, mainly leukocytes that leak from capillaries into the wound) and resident cells (cells that are present and formed in damaged tissues, mainly fibroblasts). Subsequently, during light microscopy, all preparations were divided into three areas of equal length (center of wounds and two edges). On histological preparations, the number of resident (fibroblast) and non-resident (leukocyte) cells was determined by their morphological characteristics in granulation layers in the center and both edges of wounds (Figure 1). 

The absolute number of cells (number of cells per mm^2^) and the percentage of resident and non-resident cells in different areas of the wound were analyzed. Descriptive microscopy at ×40, ×100, and ×400 and ×1000 magnifications were performed using a Levenhuk D740 microscope (USA). On preparations stained with hematoxylin–eosin, special attention was paid to the assessment of wound epithelialization (localization and length of the epithelium, the number of its layers).

Statistical analysis of the research results was carried out with the SPSS 23.0 software (IBM Corp., Armonk, NY, USA). The normal distribution of the results was checked by the Kolmogorov–Smirnov test. Descriptive statistics of continuous quantitative data with a normal distribution are presented in the form of mean and standard deviation. Descriptive statistics of quantitative data with non-normal distribution are presented as median (Me) and interquartile range (IQR), values of the lower (25%) and upper (75%) quartiles. Qualitative data were presented in the form of a percentage. The Kruskal–Wallis test was used to compare more than two independent nonparametric samples. In these cases, one-way ANOVA was used to simultaneously compare the indicators of 4 groups (independent parametric samples), followed by an appropriate post hoc analysis (Dunnett’s *t*-test for comparison with a control group) after receiving the ANOVA test of significance at *p* < 0.05. To compare two independent nonparametric samples, we used the Mann–Whitney U test. To compare two dependent nonparametric samples (dynamics of indicators in one group), we used the Wilcoxon test. Differences were considered significant at *p* < 0.05.

Ethics. The experiment was carried out in accordance with the principles of handling laboratory animals and in compliance with the provisions of the European Convention for the Protection of Vertebral Animals Used for Experimental and Other Scientific Purposes. CETS 123. The study was approved at a meeting of the Regional Ethics Committee of the Federal State Budget Educational Institution of Higher Education at the Kursk State Medical University of the Ministry of Health of Russia (Protocol No. 5 from 2 November 2017).

## 3. Results

### 3.1. The Dynamics of Wound Areas

During the study, a positive therapeutic effect of polymer nano-drugs on the dynamics of wound area reduction was established. This was most typical for the wounds of the SPN + SC group.

The comparison of the area index of all four groups simultaneously according to the Kruskal–Wallis test revealed a statistically significant difference on day 1 (*p* = 0.001), day 3 (*p* = 0.048), day 5 (*p* = 0.005), day 7 (*p* = 0.001), and day 14 (*p* = 0.0001), that is, at all control points of the study, except for day 0 (*p* = 0.204). It actualizes the pairwise comparative analysis of this indicator in dynamics.

**Figure 2:**
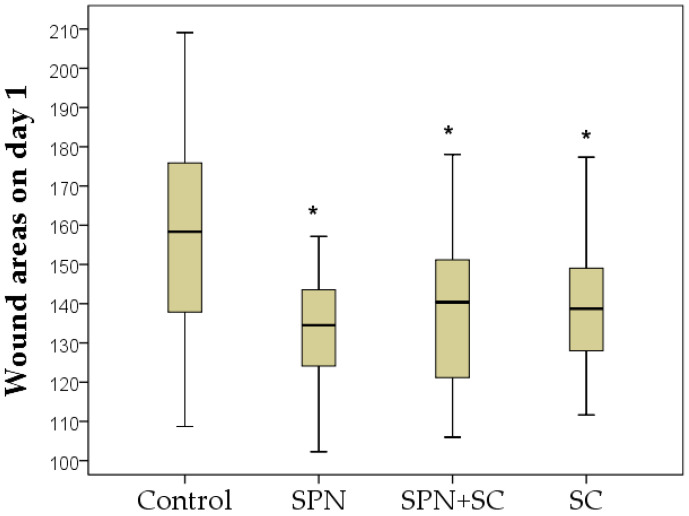
Wound areas on day 1 of the study in different groups (mm^2^). Notes: *—difference from the control group at *p* < 0.05. Post hoc analysis score (Dunnett’s *t*-test for comparison with a control group). Data are presented as median and interquartile range.

The sizes of the wounds 24 h after their modeling were the largest in the control group. The area of these wounds significantly increased in comparison with day 0 by an average of 1.2 times (*p* < 0.01). The area of wounds in the remaining groups did not statistically change compared to day 0. As a result, on day 1, statistically significant differences in the wound area of all drug groups compared to the control group were established. The size of wounds in the control group was, on average, 1.17 times greater than in the SPN group (*p* < 0.01), 1.13 times greater than in the SPN + SC group (*p* < 0.01), and 1.14 times greater than in the SC group (*p* < 0.01). The three treatment groups did not differ in this indicator on day 1 of the study (Figure 2).

**Figure 3:**
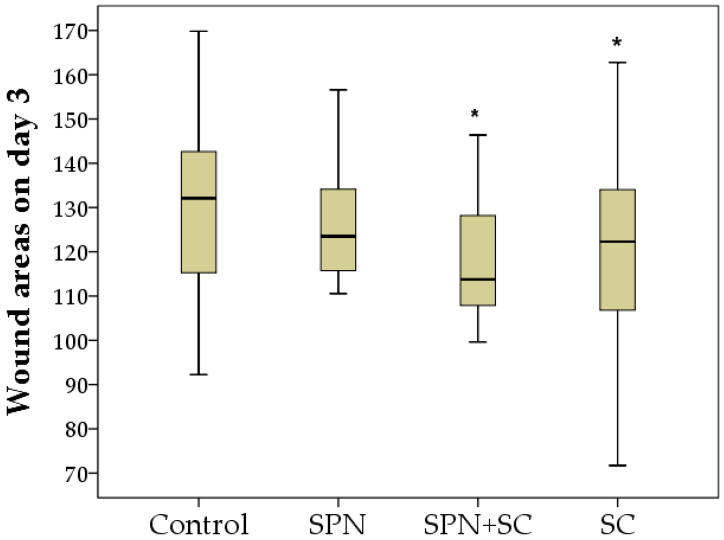
Wound areas on day 3 of the study in different groups (mm^2^). Notes: *—difference from the control group at *p* < 0.05. Post hoc analysis score (Dunnett’s *t*-test for comparison with a control group). Data are presented as median and interquartile range.

On the third day of the study, the wounds for the treatment in which stem cells were used, in particular SPN + SC, were reduced the most (the area of these wounds significantly decreased on average by 1.25 times relative to day 0, *p* < 0.01), becoming on average 113.8 mm^2^. The area of wounds in the SC and SPN groups by the end of the three days decreased by an average of 1.10 times (*p* < 0.05). The sizes of wounds in the control group were the largest (Me = 132.1 mm^2^) and did not statistically differ from day 0. The area of wounds in the control group was, on average, 1.07 times larger than in the SPN group (*p* > 0.05), 1.16 times larger than in the SPN group + SC (*p* < 0.05), and 1.08 times larger than in the SC group (*p* < 0.05) (Figure 3). In addition, it was found that the wounds of the SPN group were 1.08 times larger than the wound areas of the SPN + SC group (*p* < 0.05). This may be due to the addition of the positive therapeutic effects of SPN and SC, which were clinically noticeable by the end of the three days.

**Figure 4:**
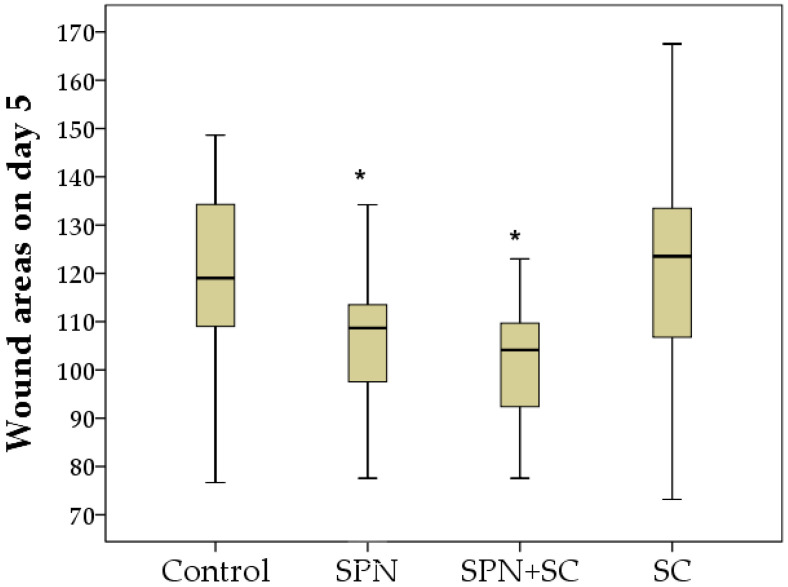
Wound areas on day 5 of the study in different groups (mm^2^). Notes: *—difference from the control group at *p* < 0.05. Post hoc analysis score (Dunnett’s *t*-test for comparison with a control group). Data are presented as median and interquartile range.

On day 5, the largest wounds were observed in the control and SC groups, which did not differ from each other in terms of the wound area. This may indicate a limited lifespan of cells injected into the wound (the effect is limited to the first 3–4 days). The use of smart polymers made it possible to improve wound healing by five days. The sizes of wounds in the control and SC groups on day 5 were, on average, 1.10 times and 1.14 times greater than in the SPN group, respectively (*p* < 0.05), and greater than in the SPN + SC group by 1.13 times and 1.17 times, respectively (*p* < 0.05). The SPN and SPN + SC groups did not differ in terms of wound area on day 5 (Figure 4).

**Figure 5:**
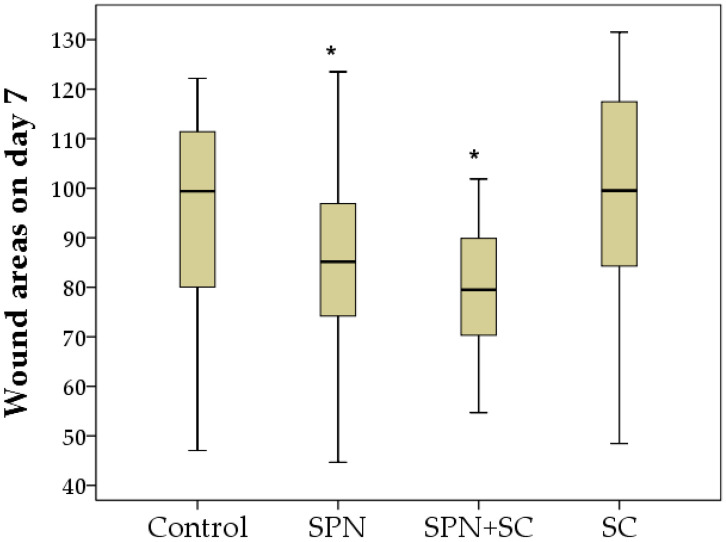
Wound areas on day 7 of the study in different groups (mm^2^). Notes: *—difference from the control group at *p* < 0.05. Post hoc analysis score (Dunnett’s *t*-test for comparison with a control group). Data are presented as median and interquartile range.

On the seventh day, as well as on the fifth day, the wound areas of the control and SC groups were the largest. The areas of wounds treated with polymer nano-drugs were smaller. The wounds of the SPN + SC group decreased by the greatest amount (1.79 times relative to day 0, *p* < 0.01). SPN group wound areas decreased 1.59 times (*p* < 0.01), SC group wound areas decreased 1.35 times (*p* < 0.01), and the control group wound areas decreased by an average of 1.32 times in size, relative to day 0 (*p* < 0.01). The size of wounds in the control group averaged 99.4 mm^2^ (IQR: 80–111.4 mm^2^), in the SPN group 85.2 mm^2^ (IQR: 74.2–96.9 mm^2^), in the SPN + SC group 79.5 mm^2^ (IQR: 70.3–89.9 mm^2^), in the SC group 99.5 mm^2^ (IQR: 84.3–117.5 mm^2^). As a result, the wound area of the SPN and SPN + SC groups was significantly smaller than in the control and SC groups. Thus, the wound size of the control and SC groups on day 7 was, on average, 1.17 times greater than in the SPN group (*p* < 0.05), and also 1.25 times greater than in the SPN + SC group (*p* < 0.01). The SPN and SPN + SC groups did not differ from each other on day 7, despite the fact that the wound area in the SPN group was, on average, 1.07 times greater than in the SPN + SC group (*p* > 0.05) (Figure 5).

Thus, the dynamics of the wound areas during the first week were as follows. By the end of the first day, wound areas were smaller in all drug groups compared to the control; by the third day, the wound areas of the SC and SPN + SC groups were smaller, and on the fifth to seventh days, the wound areas of the SC group did not differ from the wound areas of the control group, while the wounds healed better in the SPN and SPN + SC groups. 

**Figure 6:**
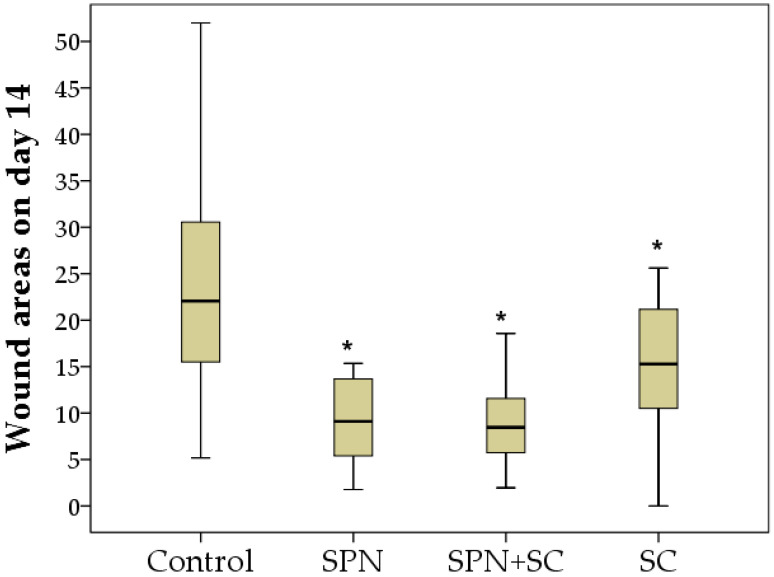
Wound areas on day 14 of the study in different groups (mm^2^). Notes: *—difference from the control group at *p* < 0.05. Post hoc analysis score (Dunnett’s *t*-test for comparison with a control group). Data are presented as median and interquartile range.

Despite the absence of treatments for all wounds in the second week of the study, statistically significant differences in the wound areas of the control group compared to the other three groups were established by the 14th day, and the best result was achieved in the SPN + SC group. The average wound area in the control group was 22.1 mm^2^; in the SC group, it was 15.3 mm^2^; in the SPN group, 9.1 mm^2^; in the SPN + SC group, 8.5 mm^2^. On the seventh day, the wound area of the control group was, on average, 2.43 times greater than in the SPN group (*p* < 0.01), 2.60 times greater than in the SPN + SC group (*p* < 0.01), and 1.44 times greater than in the SC group (*p* < 0.05). In addition, it was revealed that the wound areas of the SC group were statistically significantly larger (1.68 times) than the wound areas in the SPN group (*p* = 0.056) and 1.80 times larger than the wound areas in the SPN + SC group (*p* < 0.05) (Figure 6).

**Figure 7:**
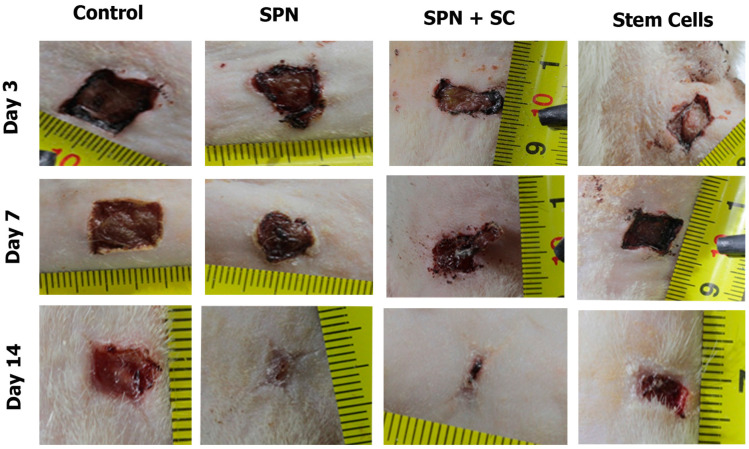
The visual dynamics of wounds in different groups.

The visual dynamics of wounds in different groups are presented in Figure 7.

### 3.2. Microcirculation Dynamics

**Figure 8:**
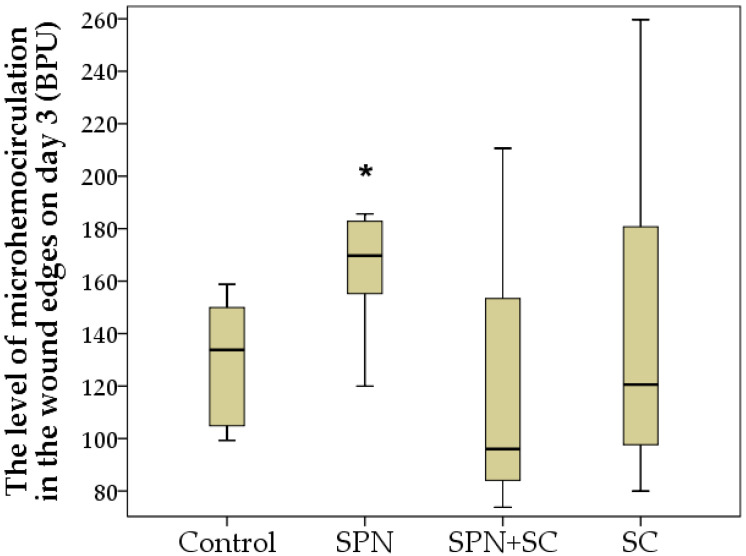
The level of microhemocirculation in the wound edges in different groups on day 3 (BPU). Notes: *—difference from the control group at *p* < 0.05. Post hoc analysis score (Dunnett’s *t*-test). Data are presented as median and interquartile range.

During the microcirculation study, statistically significant differences between the groups were established on the third day of the study. The highest level of hemoperfusion at the edge of the wounds during this period was recorded in the SPN group (Me: 169.7 BPU, IQR: 155–183 BPU). The smallest level of hemoperfusion at the edge of the wounds was recorded in the groups where stem cells were used for treatment, especially in the SPN + SC group (Me: 96 BPU, IQR: 84–153 BPU) (Figure 8). This may be due to the anti-inflammatory effect of stem cells, which is greatest in the first 3–5 days after injury. On average, on day 3 of the study, the level of microcirculation in the SPN + SC group was 1.27 times higher than in the control group (*p* < 0.05). In some cases, the use of SPN without cells created a local irritant stimulating effect with an increase in the level of microcirculation at the edge of the wounds; as a result, the wound healing process was improved. 

**Figure 9:**
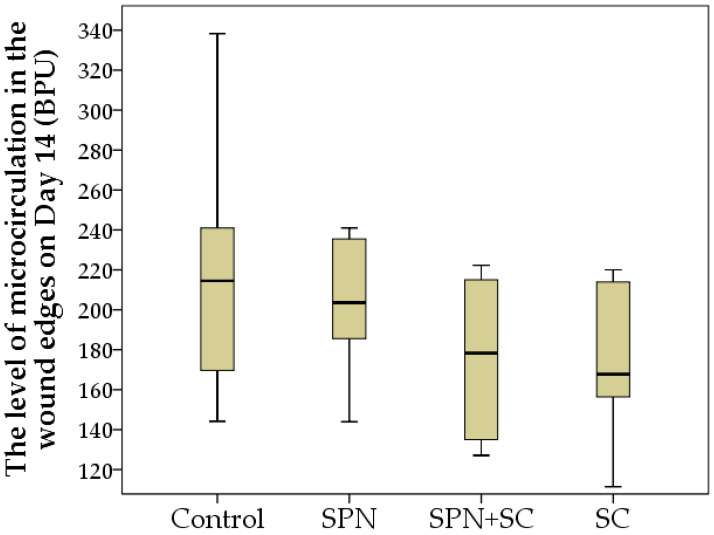
The level of microhemocirculation in the wound edges in different groups on day 14 (BPU). Notes: Data are presented as median and interquartile range.

On the seventh day, the same tendencies remained, but due to the weakening effect of the cell therapy, differences between the groups in terms of microhemocirculation were absent. On average, this indicator in different groups on day 7 was 158–189 BPU (*p* > 0.05). By the 14th day of the study, an insignificant tendency for the increased perfusion at the edge of the wounds of the control and SPN groups over the perfusion level of the SC and SPN + SC groups was noted (Figure 9).

### 3.3. Morphometry and Microscopy of Wounds

In the study of histological preparations, it was found that non-resident cells predominated in the wounds on the third day. This is due to the exudative phase of inflammation, characteristic of this period of regeneration. During this period, leukocyte cells emerging from the vessels were concentrated in the wound. Moreover, there were more leukocytes on both edges of the wounds than in the center of the wounds in all the studied groups. At the same time, the largest number of leukocytes on day 3 was observed in the control group (on average, 64% in the center and 77% at the edges of the wounds). The lowest number of leukocytes was recorded in the SC and SPN + SC groups, probably due to the anti-inflammatory effect of mesenchymal SC. The average content of non-residents in the center of wounds in these groups was the same (62%). At the edges of the wounds, there were fewer leukocytes in the SPN + SC group (71%), but not in the SC group (75%). It can be assumed that the polymer-drug potentiates the anti-inflammatory effect of stem cells and thereby improves regeneration (Table 1).

**Figure 10:**
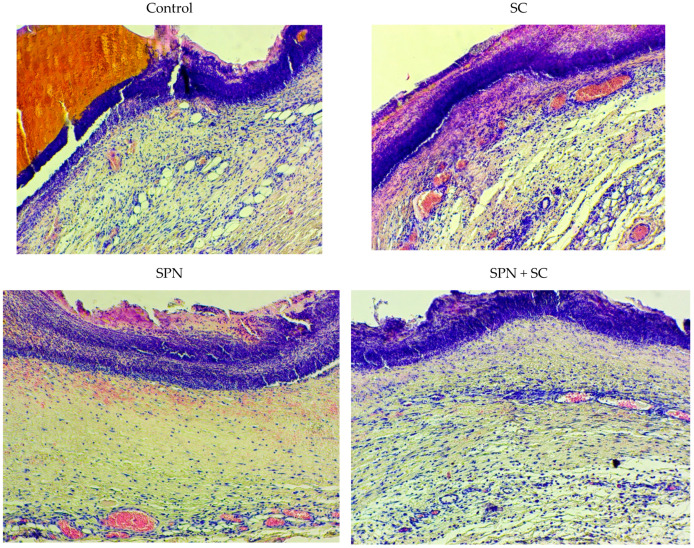
Center of wounds in different groups on the third day of the study (staining with Hematoxylin–Eosin, magnification ×100).

A decrease in leukocyte infiltration in the treatment groups was associated with the most rapid transition from exudation to proliferation. This was confirmed by the following histological signs. First, on day 3, all wounds were covered with a scab, which was soaked in polymorphonuclear leukocytes. The scab was thicker in the control group and thinner in the SC and SPN + SC groups. Second, the leukocyte infiltrates spread widely into the granulation tissue. This was most characteristic of the control group. Thirdly, in the tissues of all wounds, signs of a venous plethora of hypodermis were found, as well as the phenomenon of “marginal pool of leukocytes”. This phenomenon manifested itself in the accumulation of leukocytes in the parietal layer of the blood of postcapillary venules. This indicates the continuation of the migration process of leukocytes into the paravasal tissue, which is typical for the stage of exudation of inflammation. The lowest degree of edema and marginal leukocyte pool was recorded in the SPN + SC group (Figure 10).

On day 7, resident cells predominated in the wounds. By this time, exudative inflammation was replaced by a proliferative phase. At this point, the fibroblasts producing the extracellular matrix were the most active. The highest concentration of fibroblasts was observed in the groups using polymer drugs. This mainly concerned the central part of the wounds. This correlated with the onset of the marginal epithelialization in all groups. The highest content of fibroblasts in the center of wounds was in the SPN + SC group (on average 64%), with slightly smaller in the SPN group (62%). The lowest concentration of residents was in the control group (56%). The mean fibroblast concentration in the SC group did not differ from the control wounds (60%, *p* > 0.05), apparently due to the expiration of the SC duration (Table 2). The presence of significant differences between both polymer groups from the group control by the content of resident and non-resident cells allows us to conclude that the polymers enhanced the proliferation and regeneration of wounds on the seventh day. In addition, the data obtained suggest an increase in the lifespan of stem cells upon their integration into a nanopolymer compound of polymers with cerium dioxide.

**Figure 11:**
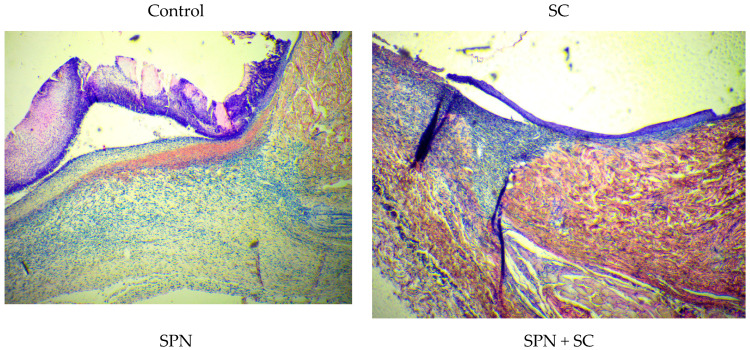

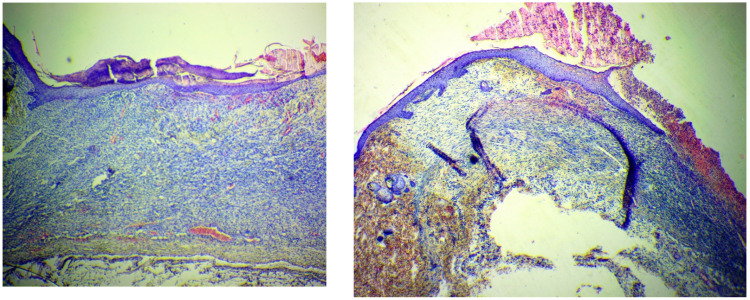
Edge of wounds in different groups on day 7 (Hematoxylin–Eosin, magnification ×40).

At the edges of the wounds, there were 1.2–1.4 times more resident cells than in the center of the wounds. Epithelialization correlated with this process. In all wounds, epithelialization was marginal. The greatest difference in the content of resident cells in the center and at the edges of wounds was in the control group, where epithelialization was the smallest. Thus, microscopy revealed that, in the edges of the wounds of the control group on day 7, signs of epidermal regeneration were the least pronounced. This was evidenced by the length of the newly formed epidermis (from the edges of the wound to the center), as well as the smallest number of layers of the epidermis in the control group. The best epithelialization (greater length and number of layers, including the superficial stratum corneum) was in the SPN and SPN + SC groups (Figure 11).

On the 14th day of the study, regional differences in the content of both resident and non-resident cells were not recorded in all groups. In the center and on both edges of the wounds, the cell concentration was comparable. This may be due to the restoration of the depth of damage by increasing the epithelialization of most wounds. The predominance of fibroblasts over leukocytes during this period demonstrates the continuing phase of proliferation during this period (Table 3).

The general analysis of the number of cells on histological preparations showed that, on the third day, when leukocytes prevailed, the number of cells was lower than at other times. At the same time, on day 3, no differences were found between the groups for this indicator. The maximum number of cells in all groups was on day 7; however, this was mainly in the SPN + SC group. The number of cells in this group increased 3.7 times as compared with three days (on average, from 247 to 919 cells per mm^2^, *p* < 0.05). In second place in terms of this indicator, were the wounds of the SPN group, where, comparing day 3 to day 7, the number of cells increased 3.0 times on average from 285 to 854 (*p* < 0.05). In the remaining groups, the number of cells increased 2.4 times (control) and 2.6 times (SC group). Significant differences were revealed, demonstrating that, in the SPN + SC group, cell infiltration was greater than in the SC and control groups (*p* < 0.05). A significant difference was also found between the SPN and SC groups (*p* < 0.05). Consequently, it was precisely the smart polymer nano-drugs that were able to activate the proliferative phase and the activity of fibroblasts in the best way by the seventh day of regeneration.

**Figure 12:**
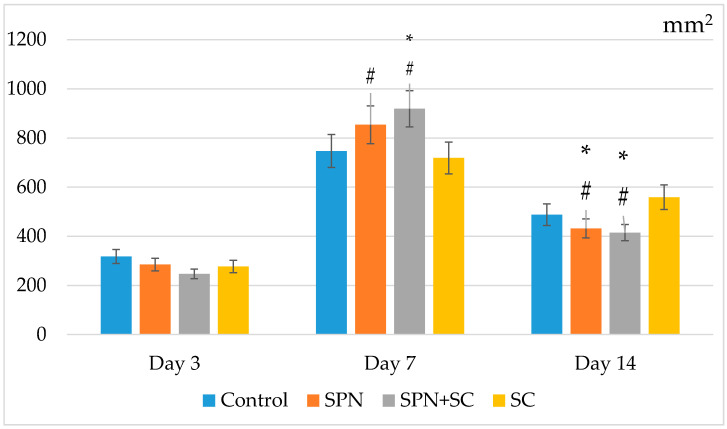
The number of resident and non-resident cells (total amount on average for wounds of different groups). Note: *—Difference from the control group at *p* < 0.05; ^#^—Difference from the SC group at *p* < 0.05 (Mann–Whitney U test). Mean and standard deviation are presented.

On the 14th day, the total number of cells of all granulation layers decreased in all groups, but the maximum decrease was recorded in the groups using polymers (on average, 2.2 times to 415 cells per mm^2^ of wound center in the SPN + SC group; 2.0 times up to 488 cells per mm^2^ wound in the SPN group). In the control group, the regression of the number of cells was 1.5 times and, in the SC group, this was 1.3 times. Statistically significant differences were found, showing that, in both polymer groups, the number of cells was lower than in the control and SC groups (Figure 12). This correlated with the best clinical and morphological wound healing results.

## 4. Discussion

Thus, the effectiveness of the use of SPN and SPN + SC for the treatment of wounds has been determined. The results obtained are consistent with the opinion of earlier studies on the effective use of polymers [10,11,12,13,14,24] and molecules of cerium dioxide [20,21,22,23,24,25] for the treatment of wounds. In addition, our study confirms the results of other studies that the introduction of nanoparticles of certain substances radically changes the effectiveness of drugs, and this represents a quantum leap in providing safe and effective targeted drug delivery to the affected tissues [31,32].

In our study, a positive therapeutic effect of polymer drugs on the dynamics of wound healing was established, which was strongly pronounced in the SPN group and particularly in the SPN + SC group. The SC-treated group of wounds failed to achieve the same results. Perhaps this is due to the single injection of cellular material and the short life span of SC introduced into animal tissues.

On the third day of the study, an anti-inflammatory effect was revealed in the SC, particularly in the SPN + SC group, which expressed lower leukocyte infiltration of wounds and an increase in the level of microcirculation during this period. This can be attributed to the proven anti-inflammatory effect of stem cells [33,34,35,36,37]. In control wounds, on the contrary, prolongation of active inflammation was recorded. This was outwardly manifested by a significant increase in the size of the wounds one day after their modeling and a decrease in the size of the wounds only from five days. Histologically, this was manifested by a more pronounced leukocyte infiltration of wound tissues and a lower content of fibroblasts, which confirmed a delay in the transition from the phase of exudation to proliferation.

At the same time, the most rapid transition from the phase of exudation to proliferation, which equates to the fastest transition to tissue repair itself, was recorded in the SPN and SPN + SC groups. Histologically, these groups have shown more rapid regeneration, including the epithelialization of the wound surface, as assessed by the length and number of layers of stratified squamous epithelium.

The use of SC gives a biological impetus to the beginning of wound regeneration. Thus, the time for preparing the cells for the beginning of the productive synthesis of the intercellular substance is shortened. In addition, the introduction of SA reduces the negative effects of the inflammation process on the rate of regeneration. It is highly likely that bacterial contamination of the wound would not achieve this effect.

The results obtained make it possible to assume success when using drugs based on polymers with integrated cerium dioxide nano-molecules. The SPN used in our study was proven to be no less effective than biological therapy using stem cell culture, the positive effect of which has been determined in many studies [26,38,39,40,41].

The maximum positive effect of SPN + SC combination therapy raises many new questions. Cell therapy probably provided an impetus for accelerated regeneration and for the best subsequent effect of SPN. This may be due to an increase in the life span of cells when they are administered together with a polymer nano-drug. However, this assumption requires further research. Moreover, significant differences in the wound areas between the SPN and SPN + SC groups were registered only on the third day, from the period of the transition of the exudative phase of inflammation to the proliferative one. To date, we cannot say whether this is due to the polymer base of the hydrogel, or to cerium dioxide. However, the data obtained allow us to assume the prospect of creating not only an effective non-invasive medicine for wound healing, but also compounds that can prolong the life of cells, biological objects, and therefore, ensure an increased shelf life of “living” combined preparations.

However, definitive answers to the newly posed questions require further studies, both in vivo and in vitro, with an increase in the observation period and the number of indicators characterizing wound healing.

The main conclusion of the original study.

1. The results obtained prove the high efficiency of drugs using nanomaterials integrated into a polymer matrix.
2. The results of the use of smart polymeric drugs indicate the need or the possibility of creating fundamentally new, highly effective drugs.
3. The drugs developed and studied in the manuscript can be used in clinical practice for the treatment of acute and long-term non-healing chronic wounds.

## Figures and Tables

**Table 1:** The content of resident and non-resident cells in the center and edges of wounds of different groups on day 3 (%).

Groups	Residents on Day 3			Non-Residents on Day 3		
	Edge 1	Center	Edge 2	Edge 1	Center	Edge 2
Control	21.8 ± 4.6	35.6 ± 2.6	23.4 ± 6.4	78.2 ± 4.6	64.4 ± 2.6	76.6 ± 6.4
SPN	24.6 ± 5.2	37.0 ± 4.5	22.1 ± 6.1	75.4 ± 5.2	63.0 ± 4.5	77.9 ± 6.1
SPN + SC	35.8 ± 10.1	37.9 ± 4.7	21.4 ± 9.9	64.2 ± 10.1	62.1 ± 4.7	78.6 ± 9.9
Stem Cells	26.3 ± 5.1	37.7 ± 4.9	22.5 ± 6.4	73.7 ± 5.1	62.3 ± 4.9	77.5 ± 6.4

Edge 1 is caudal (localized closer to the tail), edge 2 is head. Resident cells—fibroblasts that are present and formed in damaged tissues. Non-resident cells—leukocytes that leak from capillaries into the lesion focus.

**Table 2:** The content of resident and non-resident cells in the center and edges of wounds of different groups on day 7 (%).

	Residents on Day 7			Non-Residents on Day 7		
Groups	Edge 1	Center	Edge 2	Edge 1	Center	Edge 2
Control	76.1 ± 3.9	56.4 ± 5.9	72.2 ± 7.1	23.9 ± 3.9	43.6 ± 5.9	27.8 ± 7.1
SPN	77.4 ± 4.0	62.0 ± 3.4 *	72.3 ± 6.5	22.6 ± 4.0	38.0 ± 3.4 *	27.7 ± 6.5
SPN + SC	79.0 ± 4.4	63.7 ± 2.3 *	72.5 ± 6.1	21.0 ± 4.4	36.3 ± 2.3 *	27.5 ± 6.1
Stem Cells	75.7 ± 3.0	60.3 ± 3.0	71.5 ± 4.4	24.3 ± 3.0	39.7 ± 3.0	28.5 ± 4.4

Notes: *—Difference from the control group at *p* < 0.05 (Mann–Whitney U test). Mean and standard deviation are presented. Edge 1 is caudal (localized closer to the tail), edge 2 is head.

**Table 3:** The content of resident and non-resident cells in the center and edges of wounds of different groups on day 14 (%).

Groups	Residents on Day 14			Non-Residents on Day 14		
	Edge 1	Center	Edge 2	Edge 1	Center	Edge 2
Control	72.8 ± 5.4	72.6 ± 6.0	80.4 ± 4.9	27.2 ± 5.4	27.4 ± 6.0	19.6 ± 6.0
SPN	76.3 ± 3.0	71.5 ± 4.9	77.2 ± 4.3	23.7 ± 3.0	28.5 ± 4.9	22.8 ± 4.3
SPN + SC	77.1 ± 2.8	69.4 ± 5.4 *	76.7 ± 5.5	22.9 ± 2.8	30.6 ± 5.4 *	23.3 ± 5.5
Stem Cells	75.6 ± 3.1	75.2 ± 4.1	78.0 ± 3.1	24.4 ± 3.1	24.8 ± 4.1	22.0 ± 3.1

Notes: *—Difference from the control group at *p* < 0.05 (Mann–Whitney U test). Mean and standard deviation are presented. Edge 1 is caudal (localized closer to the tail), edge 2 is head.

## Data Availability

The data presented in this study are available on request from the corresponding author.
